# Diagnosis and treatment of bilateral adrenal pheochromocytoma with RET gene mutation combined with medullary sponge kidney: A case report

**DOI:** 10.1097/MD.0000000000034022

**Published:** 2023-06-09

**Authors:** Pengliang Shen, Nan Yin, Libin Sun, Yunfeng Liu, Xiaoming Cao

**Affiliations:** a Department of Urology, First Hospital of Shanxi Medical University, Taiyuan, Shanxi, China; b Department of Endocrinology, First Hospital of Shanxi Medical University, Taiyuan, Shanxi, China.

**Keywords:** adrenal gland, medullary sponge kidney, pheochromocytoma, RET proto-oncogene

## Abstract

Pheochromocytomas are a group of tumors with high genetic heterogeneity, and the clinical characteristics of rearranged during transfection (RET)-mutated pheochromocytoma with medullary spongiform kidney are rarely studied. The treatment process of 1 patient with bilateral adrenal pheochromocytoma combined with medullary sponge kidney with RET gene mutation in our department was retrospectively analyzed, and the treatment methods for this type of disease were studied and summarized in combination with relevant literature.

**Patient concerns:**

In this case, the patient was found to have bilateral adrenal masses for 8 years due to physical examination, and intermittent dizziness and discomfort for 2 years. Imaging and related laboratory examinations suggest bilateral adrenal giant pheochromocytoma with bilateral medullary sponge kidney. RET gene testing was performed on the patient and his descendant after signing the informed consent form.

**Diagnoses:**

The patient was diagnosed with bilateral adrenal pheochromocytoma with a RET proto-oncogene mutation and a bilateral medullary spongy kidney.

**Intervision and outcomes:**

After sufficient perioperative preparation, retroperitoneal laparoscopic bilateral adrenal pheochromocytoma resection was performed by stages. The operation was successful, and hormone replacement therapy was performed after the operation, with regular follow-up. Relevant genetic testing revealed that the c.1900T > C: p.C634R mutation was detected in the patient’s RET gene, which was a heterozygous missense mutation, and the mutation was also present in the son of his family. A literature analysis found that pheochromocytoma is a tumor with high genetic heterogeneity, and the RET proto-oncogene is a common pathogenic gene for bilateral adrenal pheochromocytoma. Medullary sponging of kidneys is a rare complication of this disease.

**Lessons:**

On the basis of adequate perioperative preparation, surgical resection is the most effective and preferred treatment for this type of disease. Laparoscopic surgery is minimally invasive, safe, and effective by stages. Mutations in the RET proto-oncogene may lead to medullary spongy kidneys in multiple endocrine neoplasia 2.

## 1. Introduction

Adrenal pheochromocytoma originates from the chromaffin cells in the adrenal medulla. It mainly synthesizes and secretes a large amount of catecholamines, causing a series of clinical symptoms, such as elevated blood pressure in patients, which can be life-threatening in severe cases. Adrenal pheochromocytomas are mostly unilateral, but hereditaries are mostly bilateral and multiple, such as multiple endocrine neoplasia 2 (MEN2), in which 50% to 80% of patients are bilateral.^[1]^ Owing to the specificity of the synthesis and secretion of large amounts of catecholamines in bilateral adrenal pheochromocytomas, critical situations are prone to occur during clinical diagnosis and treatment, posing a significant threat to the safety of patients. Therefore, it is important to improve our understanding of this treatment process. Rearranged during transfection (RET) proto-oncogene is a common pathogenic gene in pheochromocytoma/paraganglioma.^[2,3]^ The clinical characteristics of RET-mutated pheochromocytomas with medullary sponge kidneys have rarely been studied. This article analyzes the clinical data related to the diagnosis and treatment process of 1 patient admitted to our department and combines the literature to conduct literature studies on the diagnosis and treatment methods of this type of disease to further improve the diagnosis and treatment of this type of disease.

## 2. Case presentation

**Figure 1.**
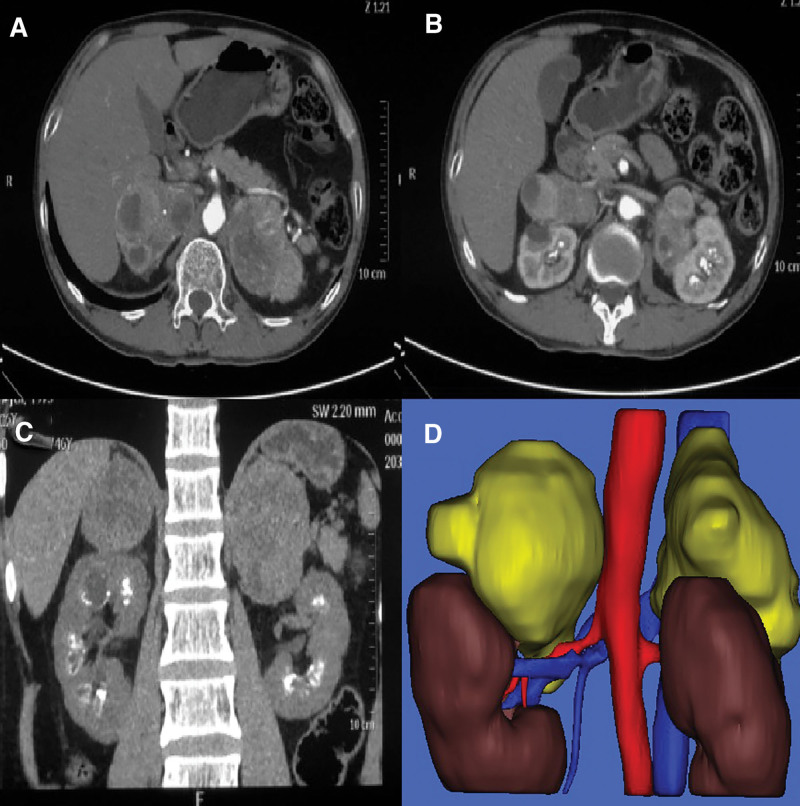
Preoperative imaging of bilateral adrenal pheochromocytoma with medullary sponge kidney. (A and B) are enhanced CT image of the adrenal gland (bilateral adrenal regions have large masses, uneven enhancement, and necrosis of central tissue), (C) is a coronal image of CT scan (multiple scattered or clustered calcifications can be seen in the renal vertebral body, and irregular low-density lesions can be seen in some peripheral areas), (D) is a 3-dimensional reconstructed image. CT = computed tomography.

A 46 years old man presented to our hospital with the main reason for admission was “physical examination found bilateral adrenal gland masses for 8 years, intermittent dizziness and discomfort for 2 years”; The patient underwent physical examination 8 years ago and found bilateral adrenal masses. Subsequently, he underwent regular reexamination. Two years prior, he experienced intermittent dizziness and transient amenorrhea, and the frequency gradually increased. He sought medical advice in our hospital for further diagnosis and treatment; Have a history of “hypertension” for 18 years; The medical history of “diabetes” is 2 years. Physical examination revealed a blood pressure of 185/100 mm Hg, heart rate of 90 beats/minutes, and no obvious nodules were palpated in the bilateral thyroid regions. Computed tomography findings (Fig. 1): Bilateral adrenal pheochromocytoma (left side size:7.5 × 7.0 cm, right side size:9.0 × 8.0 cm); Bilateral medullary sponge kidney; Cystic degeneration of pituitary adenoma. Double kidney emission computed tomography indicates that total and sub-kidney functions are normal. Blood glucose:8.5 mmol/L, creatinine:90 µmol/L. Adrenal function examination showed that 24-hour urinary free adrenaline was > 248 ug/24 hours, urinary free norepinephrine was1130 ug/24 hours, urinary methoxyepinephrine was > 2480 ug/24 hours, urinary methoxynorepinephrine was > 4960 ug/24 hours. Blood free methoxy epinephrine was 3134.0 pg/mL, and blood free methoxy norepinephrine was 8946.4 pg/mL. During the preoperative examination, the thyroid, parathyroid, and other endocrine organs were examined and no significant abnormalities were found.

**Figure 2.**
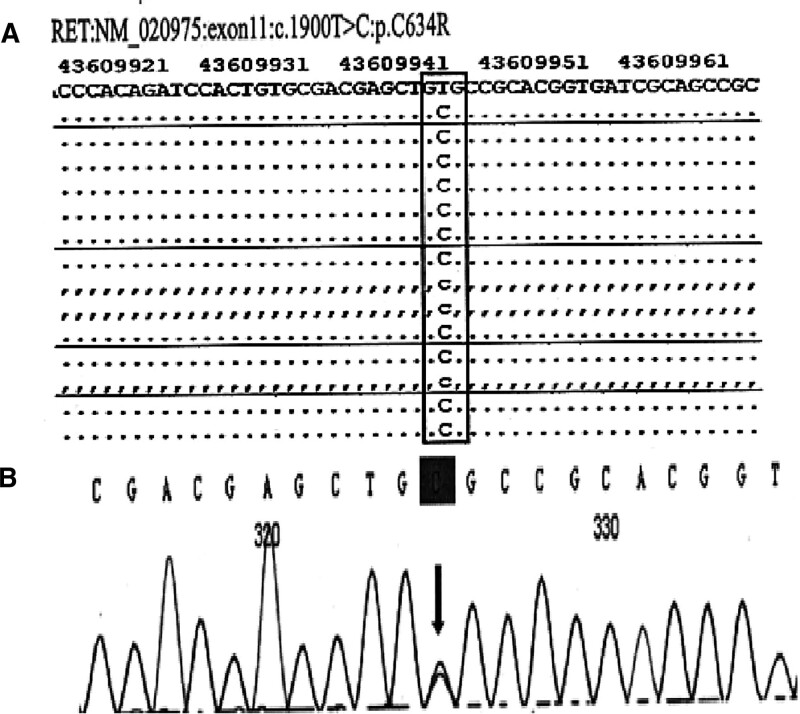
Gene sequencing results of patient. A mutation of c.1900T > C: p.C634R was detected in the RET gene, and the patient had a heterozygous missense mutation (A and B). This mutation caused the 634 codon of the RET gene encoding protein to change from cysteine to arginine. RET = rearranged during transfection.

**Figure 3.**
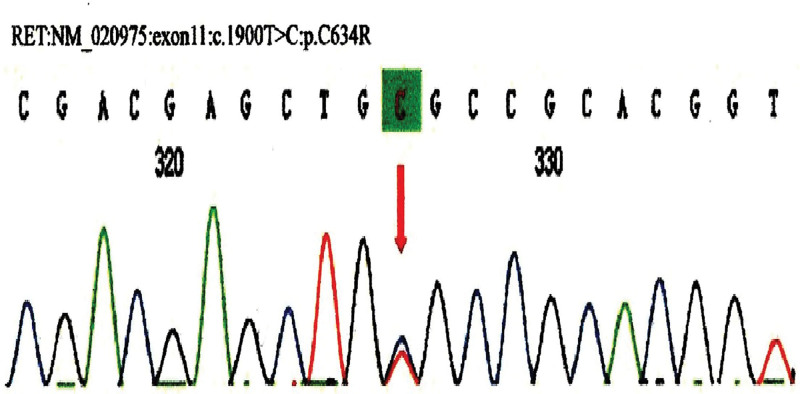
Gene sequencing results of the patient’s offspring. The offspring also had a c.1900T > C: p.C634R mutation in the RET gene. RET = rearranged during transfection.

Considering the possibility of multiple endocrine adenoma syndrome, RET gene testing was performed on the patient and his descendant after signing the informed consent form. Using target region capture and high-throughput sequencing technology, mutations in the exons of related genes and their adjacent ± 10 bp intron regions (including point mutations, deletions, and insertions within 20 bp) were identified, and the data were interpreted in accordance with the relevant guidelines of the American College of Medical Genetics and Genomics. A heterozygous missense mutation c.1900T > C: p.C634R was found in the RET gene of the subject, which resulted in a change in 634 codon of the RET gene from encoding cysteine to arginine. The results of first-generation sequencing verification showed that the mutation was true and reliable in the subject; no minor genetic variation was detected, and no mitochondrial genomic pathogenic variation was detected (Fig. 2). According to the mutation sites detected in the genetic testing of this patient, a first-generation sequencing verification was performed on his immediate family (son), and the following mutations were found: RET: NM-020975: exon11: c. 1900T > C: p.C634R. Note that the variant also exists in his offspring, and it is a heterozygote of the variant (Fig. 3).

**Figure 4.**
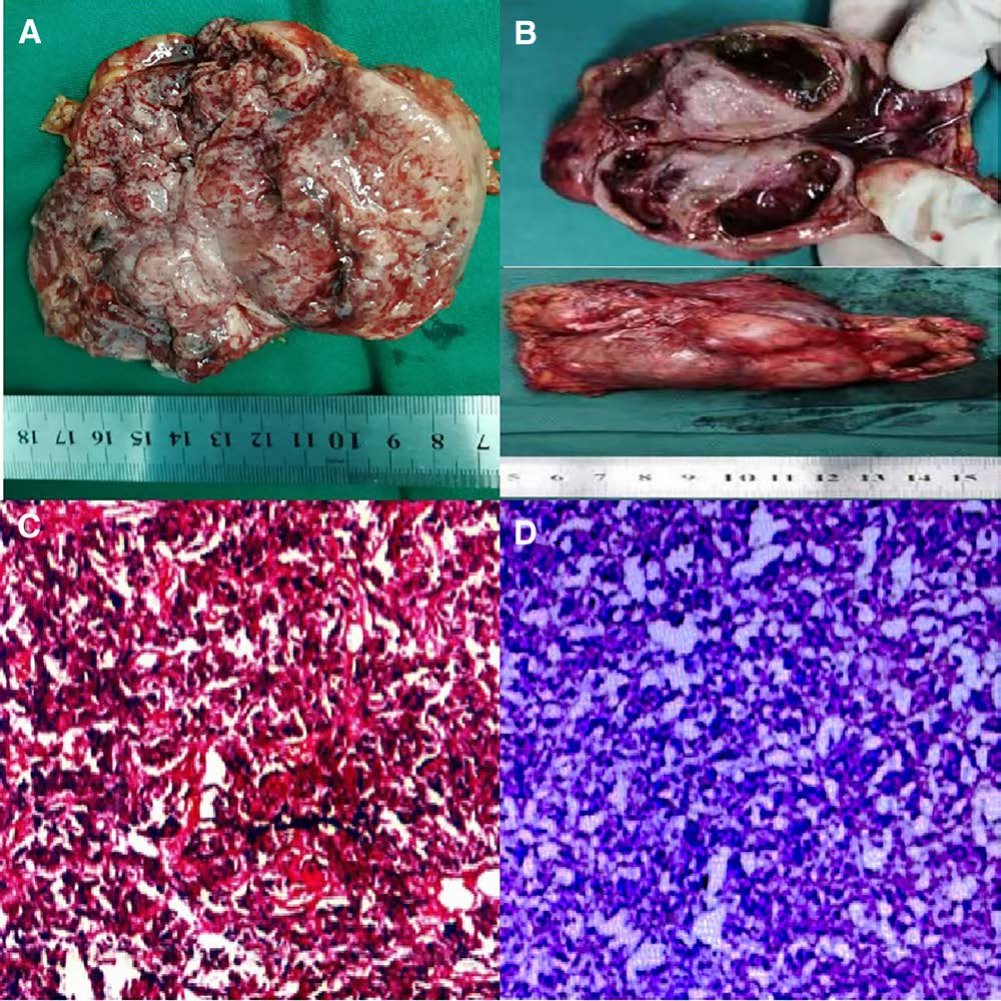
Pathological images after 2 operations. (A) The postoperative specimen of left adrenal pheochromocytoma, (B) the postoperative specimen of right adrenal pheochromocytoma, (C) a postoperative pathological picture of left adrenal pheochromocytoma (HE × 100), and (D) a postoperative pathological picture of right adrenal pheochromocytoma (HE × 100).

After the diagnosis was confirmed, we provided adequate preoperative preparation: oral α-receptor blocker: terazosin (4 mg/day), oral β-receptor blockers: metoprolol sustained-release tablets (23.75 mg/day), oral dilute saline volume expansion (1500 mL/day), intravenous infusion of supplemental colloid 1 week before surgery, and intramuscular injection of dexamethasone 3 mg at 19:00 pm on the first day before surgery, after which relevant indicators such as preoperative blood pressure, heart rate, and red blood cell volume were prepared to meet the standards, retroperitoneal laparoscopic resection of the left adrenal pheochromocytoma under general anesthesia was performed. The operation was successful, and the postoperative hormone supplementation plan was as follows: on the first day after operation, 3 mg of dexamethasone was injected intramuscularly at 07:00 in the morning, dexamethasone 3 mg intramuscular injection at 19:00 pm; On the second day after surgery, dexamethasone 1.5 mg was injected intramuscularly at 07:00 in the morning, dexamethasone 1.5 mg intramuscularly at 19:00 pm; On the 3rd day after surgery, dexamethasone 0.75 mg was injected intramuscularly at 07:00 in the morning, dexamethasone 0.75 mg intramuscular injection at 19:00 pm; On the first day after surgery, hydrocortisone sodium succinate 300 mg/24 hours + norepinephrine was continuously pumped in; The second day after surgery: hydrocortisone sodium succinate 200 mg/24 hours; On the 3rd day after surgery, hydrocortisone sodium succinate 100 mg/24 hours; In the future, it will gradually decrease gradually by taking hydrocortisone acetate tablets 20 mg/time 3 times/day, and gradually decrease according to the recovery of blood pressure, heart rate, etc. Postoperative pathological results suggest (Fig. 4): (Left adrenal) pheochromocytoma with hemorrhage and necrosis, size approximately 7.5 × 7.0 × 5.0 cm; Immunohistochemical results showed: CgA (+), Syn (+), CK (−), Vim (−), Calretin (−), Inhibin α (−), Melan-A(+), Ki67(2%+), S100(+).

**Figure 5.**
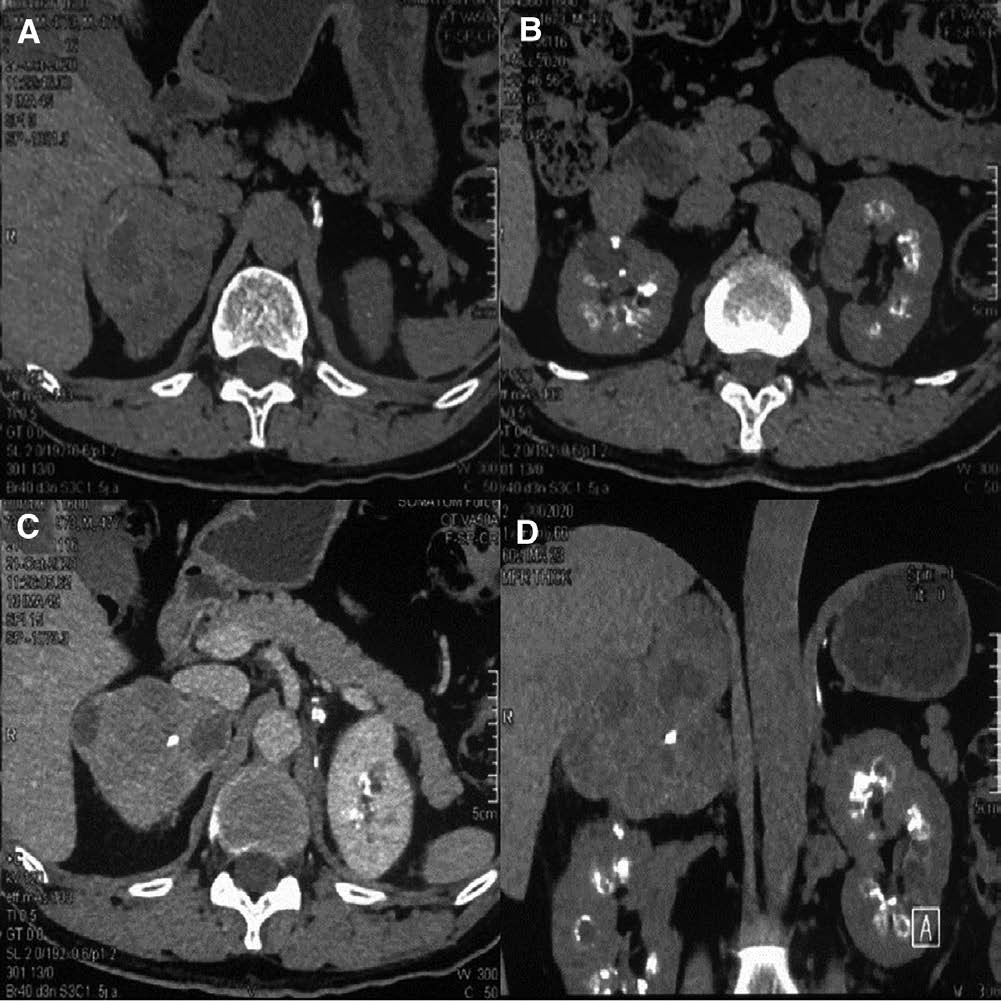
Preoperative reexamination images for the second operation. (A and B) adrenal CT plain scan images, (C) an enhanced CT image of the adrenal gland (post operation on the left adrenal gland, right adrenal pheochromocytoma), (D) a coronal CT scan image (a large mass in the right adrenal region, bilateral medullary spongy kidneys). CT = computed tomography.

After the end of this operation, the patient was prepared for 2 months using the same method described above, and the adrenal computed tomography findings were reviewed (Fig. 5): After left adrenalectomy, the right adrenal pheochromocytoma (about 9.5 × 8.5 cm), the tumor volume slightly increased compared to the previous bilateral medullary sponge kidney. The review of adrenal functional examination showed that 24-hour urinary methoxy epinephrine was 11800.0 pmol/L, urine methoxynorepinephrine was 9982.8 pmol/L; Blood free methoxy epinephrine was 3809.1 nmol/24 hours, blood free methoxy norepinephrine was 1914.6 nmol/24 hours. After preoperative preparation using the same method as the first time, retroperitoneal laparoscopic resection of the right adrenal pheochromocytoma was performed under general anesthesia. The surgery was successful, and hormone supplementation was administered using the same protocol as for the first time. Postoperative pathological results suggest (Fig. 4): Right adrenal pheochromocytoma with necrosis, the size of the tumor is about 10 × 8.5 × 4.0 cm; Immunohistochemical results showed that CK (−), CgA (+), Syn (+), Melan-A (−), Ki67 (3%+), Inhibin α (−), Vim (−/+), Calretinin (stove+), Bcl-2 (−). Since then, the patient was diagnosed with bilateral adrenal pheochromocytoma with a RET proto-oncogene mutation and a bilateral medullary spongy kidney. The patient was followed-up regularly after surgery and did not experience any of the above symptoms. Hydrocortisone acetate tablets were regularly taken orally at a dose of 20 mg per day, and thyroid ultrasound and calcitonin levels were regularly examined. The children also underwent regular endocrine system-related examinations such as adrenal color Doppler ultrasound and thyroid color Doppler ultrasound.

## 3. Discussion

Pheochromocytomas are a group of tumors with high genetic heterogeneity, with 35% to 40% of patients suffering from the disease being associated with germline mutations in known pathogenic genes. The RET proto-oncogene is a common pathogenic gene in pheochromocytoma. Patients with RET mutations often have gland tumors other than pheochromocytoma, forming the MEN2 type. MEN2 can also be divided into MEN2A and MEN2B, both of which can lead to pheochromocytoma and medullary thyroid carcinoma. MEN2A can also be accompanied by hyperparathyroidism, whereas MEN2B hyperparathyroidism is relatively rare and can be accompanied by clinical manifestations such as congenital megacolon, Marfan sign, and mucosal neuroma.^[2,4]^ Studies have shown that 80% of bilateral pheochromocytomas, mainly MEN2A (42.6%), the incidence of pheochromocytoma and medullary thyroid carcinoma in MEN2A patients is generally around 40 years of age, which is similar to the onset age of this patient. Mutations in the RET proto-oncogene and early gene testing can be detected as soon as possible,^[5]^ this patient did not have any other endocrine system-related lesions such as medullary thyroid carcinoma, which currently does not meet the relevant diagnostic criteria of MEN2A.^[1,5]^ It is possible that relevant symptoms have not yet appeared, and close observation and follow-up are still required.

Removal of bilateral adrenal pheochromocytomas may lead to decreased adrenal cortical and medullary function, resulting in complex metabolic changes that can lead to instability in the patient’s general condition, especially in the cardiovascular system, which may lead to adrenal crisis. Mild symptoms may manifest as acute anxiety syndrome, whereas severe symptoms may develop into hypotension, shock, coma, and death. Bilateral total adrenalectomy carries the risk of lifelong hormone replacement and Addison crisis in patients.^[6,7]^ Pheochromocytoma is generally characterized by a large tumor volume, rich blood supply, proximity to large blood vessels, and is prone to severe fluctuations in blood pressure during surgery. Changes in the concentration of catecholamines in blood before and after surgery can cause hemodynamic changes. The incidence of cardiovascular accidents is high.^[8]^ We administered hydrocortisone 100 mg before surgery and added 100 mg every 4 hours during the operation. The patient’s vital signs were stable during the operation. After surgery, we continued to pump “hydrocortisone” intravenously, starting from 300 mg/24 hours, and reduced the daily dose by 100 mg until the transition to the preoperative oral dose. The patient recovered smoothly, ensuring stability of the hemodynamics during the perioperative period to the greatest extent. For giant pheochromocytomas with a diameter ≥ 6 cm, most guidelines recommend open surgical resection. However, we performed laparoscopic surgery by stages, based on sufficient preoperative preparation. The surgical process was smooth, and the postoperative recovery was good. This treatment is minimally invasive, safe, and effective.

Studies have shown that mutations in the RET proto-oncogene are associated with the MEN2 type. The RET proto-oncogene encodes a transmembrane receptor of the tyrosine kinase receptor superfamily located on chromosome 10q 11.2. RET proto-oncogene undergoes mutations that cause Cys to be replaced by other amino acids and cannot be paired within the molecule. Instead, it pairs with Cys of neighboring molecules, resulting in the dimerization of RET, triggering automatic phosphorylation of tyrosine kinases and further activating the mitogen-activated protein kinase pathway, inducing excessive cell proliferation leading to canceration.^[9]^ Currently, there are multiple known mutation sites, which are commonly found in exons 10 (codons 609, 611, 618, 620) and 11 (codon 634). Codon 634 mutations are the most common, accounting for 80% of MEN2A mutations. Most patients with pheochromocytoma with RET mutations exhibit paroxysmal elevated or normal blood pressure, indicating a paroxysmal release of catecholamines in these patients, causing a paroxysmal increase in blood pressure. During the release interval, the patient’s blood and urine catecholamine levels were normal. This patient had paroxysmal hypertension, consistent with the clinical manifestation of pheochromocytoma caused by mutations in the RET proto-oncogene gene. A heterozygous missense mutation c.1900T > C: p.C634R was found in both the patient and his offspring during the gene testing. This mutation caused 634 codon of RET to change from coding cysteine to arginine, consistent with common mutation types. The mutation in the RET proto-oncogene gene of this patient is inherited in a family, and family members should closely follow-up on related diseases, early detection, and early treatment.

Bilateral adrenal pheochromocytomas with medullary spongy kidneys are extremely rare. A medullary spongy kidney is a relatively common renal developmental abnormality in clinical practice and usually manifests as renal calcinosis and recurrent renal stones. Its pathogenesis involves factors such as renal collecting duct obstruction (infectious factors are common) and abnormal embryonic development.^[10]^ Mutations in the RET proto-oncogene are not only related to MEN2 disease but also to the formation of the kidney during the embryonic period.^[11]^ Studies have shown that the RET signaling pathway plays a crucial role in kidney formation, and mutations in RET can lead to renal dysplasia. In most cases of MEN2 disease with RET gene mutations and bilateral adrenal pheochromocytomas, patients with medullary spongy kidneys have been less reported,^[12]^ and some reports suggest that mutations in the RET gene are associated with calcification and stone formation in medullary spongy kidneys,^[13]^ the specific mechanism is still unclear; only a few reports mention medullary spongy kidneys in bilateral adrenal pheochromocytoma and MEN disease with RET gene mutations.^[14,15]^ There are currently no relevant reports on the specific mechanism of medullary spongy kidney formation in bilateral adrenal pheochromocytoma, which may be related to the effect of RET gene mutations on renal development.^[12]^ In this case, the imaging examination showed that the patient had bilateral medullary spongy kidneys, and the patient’s renal function was normal; therefore, the medullary sponge kidney was not specially treated during this visit, and it was recommended to be closely followed-up. This patient had a mutation in the RET family gene and bilateral adrenal pheochromocytoma with medullary sponge kidney. However, the specific pathogenesis remains to be further studied.

## 4. Conclusion

In summary, if bilateral adrenal pheochromocytomas are found, especially when accompanied by other endocrine system-related diseases such as medullary thyroid carcinoma and parathyroid adenoma, the possibility of MEN2 should be suspected. For patients suspected of having MEN2 and their immediate relatives, genetic testing should be performed to confirm the diagnosis. Based on adequate perioperative preparation, surgical resection is the most effective and preferred treatment for this disease. Laparoscopic surgery is minimally invasive, safe, and effective by stages. Patients and relatives with genetic mutations should be closely followed-up after surgery to detect and diagnose possible concomitant diseases early. This patient was rarely accompanied by medullary spongy kidney, suggesting that mutation in the RET proto-oncogene may lead to medullary spongy kidney in MEN2; however, the specific pathogenesis remains to be further studied. At the same time, in the follow-up process in the future, it is also necessary to pay close attention to the patient’s renal function and the progress of the medullary sponge kidney, and provide early intervention if necessary.

## Acknowledgements

The authors would like to thank the supporting of the funding.

## Author contributions

**Data curation:** Pengliang Shen, Nan Yin, Libin Sun.

**Funding acquisition:** Xiaoming Cao.

**Investigation:** Pengliang Shen, Yunfeng Liu, Xiaoming Cao.

**Methodology:** Nan Yin, Libin Sun, Yunfeng Liu.

**Supervision:** Xiaoming Cao.

**Writing – review & editing:** Pengliang Shen, Xiaoming Cao.
